# Supplementary material for: Sporulation-specific cell division defects in ylmE mutants of Streptomyces coelicolor are rescued by additional deletion of ylmD
Source: Sci Rep. 2018 May 9;8:7328. doi: 10.1038/s41598-018-25782-1 (PMC5943314; doi:10.1038/s41598-018-25782-1)
Supplement: Supplementary file 1 — Supplementary Information [file 41598_2018_25782_MOESM1_ESM.pdf]

SUPPLEMENTAL INFORMATION

Belonging to the manuscript

Sporulation-specific cell division defects in *ylmE* mutants of *Streptomyces coelicolor* are rescued by additional deletion of *ylmD*

by

Le Zhang[1], Joost Willemse[1], Paul A. Hoskisson[2] and Gilles P. van Wezel[1, *]

[1] *Molecular Biotechnology, Institute of Biology, Leiden University, Sylviusweg 72, 2333 BE, The Netherlands*

[2] *Strathclyde Institute of Pharmacy and Biomedical Science, University of Strathclyde, 161 Cathedral Street, Glasgow, G4 0RE, United Kingdom*

* Author for correspondence. Tel: +31 71 5274310; email: g.wezel@biology.leidenuniv.nl.

Table S1. Bacterial strains.

Bacteria strains	Genotype	Reference
E. coli JM109	See reference	[1]
E. coli ET12567	See reference	[2]
S.coelicolor M145	SCP1⁻ SCP2⁻	[3]
K202	M145 + KF41	[4]
GAL47	M145Δ*ylmD*	This study
GAL48	M145Δ*ylmE*	This study
GAL49	M145 + pGWS757	This study
GAL50	M145 + pGWS758	This study
GAL52	GAL47 + pKF41	This study
GAL53	GAL48 + pKF41	This study
GAL128	GAL47 + pGWS1043	This study
GAL129	GAL48 + pGWS1042	This study
GAL130	M145Δ*ylmDE*::*aac(3)IV*	This study

Table S2. Plasmids and Constructs.

Plasmid and constructs	Description	Reference
pWHM3	*E. coli/Streptomyces* shuttle vector, high copy number and unstable in *Streptomyces.*	[5]
pHJL401	*E. coli/Streptomyces* shuttle vector, 5-10 copies per chromosome in *Streptomyces* and multicopy in *E. coli.*	[6]
pGWS728	pWHM3 containing flanking regions of *S. coelicolor ylmE* (SCO2080), with the apramycin resistance cassette with *loxP* sites inserted as an XbaI fragment between the flanking regions.	This study
pGWS729	pWHM3 containing flanking regions of *S. coelicolor ylmD* (SCO2081), with the apramycin resistance cassette with *loxP* sites inserted as an XbaI fragment between the flanking regions.	This study
pUWL-Cre	Plasmid expressing the Cre recombinase.	[7]
pKF41	integrative construct expressing *ftsZ-egfp* from the natural *ftsZ* promoter region.	[4]
pGWS757	pHJL401 harboring *ylmE-egfp* under control of the *ftsZ* promoter region.	This study
pGWS758	pHJL401 harboring *ylmD-egfp* under control of the *ftsZ* promoter region.	This study
pGWS1042	pHJL401 harboring the +1/+723 region of *ylmE* under control of *ftsZ* promoter	This study
pGWS1043	pHJL401 harboring the +1/+732 region of *ylmD* under control of *ftsZ* promoter	This study
pGWS1044	pWHM3 containing upstream region of *S. coelicolor ylmD*	

	(SCO2081) and downstream region of *S. coelicolor ylmE* (SCO2080), with the apramycin resistance cassette with *loxP* sites inserted as an XbaI fragment between the flanking regions.	
pKR8	pIJ8600 harboring *murD*, *ftsW*, *murG*, *ftsQ*, *ftsZ*, *ylmD*, *ylmE*, *sepF*, *sepG* and divIVA genes of *S. coelicolor*	8

Table S3. Oligonucleotides.

**Name**	**5'-3' sequence #**
ylmE_LF-1455	GTCA**GAATTC**CGTCATCCCCAACGACCGGCTGCTG
ylmE_LR+24	GTCAGAAGTTATCCATCACC**TCTAGA**GAGTTCGTGCTTACGGTCCGTCAT
ylmE_RF+697	GTCAGAAGTTATCGCGCATC**TCTAGA**GGAGTCCGACCCAGGCTCGGGTAA
ylmE_RR+2145	GTCA**GGATCC**GTGCTGCTGATCCTGCGGAGGTTC
ylmD_LF-1452	GTCA**GAATTC**GCAGCGCTACGGCTGATCACATAG
ylmD_LR+24	GTCAGAAGTTATCCATCACC**TCTAGA**CACGGTGTCGCGCTGTCCTATCAC
ylmD_RF+706	GTCAGAAGTTATCGCGCATC**TCTAGA**GCGGGCTATGTGTGGCTGGACTGA
ylmD_RR+1951	GTCA**GGATCC**TAGGGCTCTCGTTCGGACACGACC
ylmE_F+1	GTCAGAATTC**AGGCCT**TCGACATGACGGACCGTAAGCACGAACTC
ylmE_R+717	GTCAAAGCTT**GGATCC**CCGAGCCTGGGTCGGACTCCGAG
ylmE_R+723	CATG**TCTAGA**ACGTTACCCGAGCCTGGGTCG
ylmD_F+1	GTCAGAATTC**AGGCCT**TCGACGTGATAGGACAGCGCGACACC
ylmD_R+726	GTCAAAGCTT**GGATCC**TCCAGCCACACATAGCCCGC
ylmD_R+732	CATG**TCTAGA**CCATCAGTCCAGCCACACATA

Restriction sites used for cloning are shown in bold face. GGATCC, BamHI; GAATTC, EcoRI; AGGCCT, StuI; TCTAGA, XbaI.

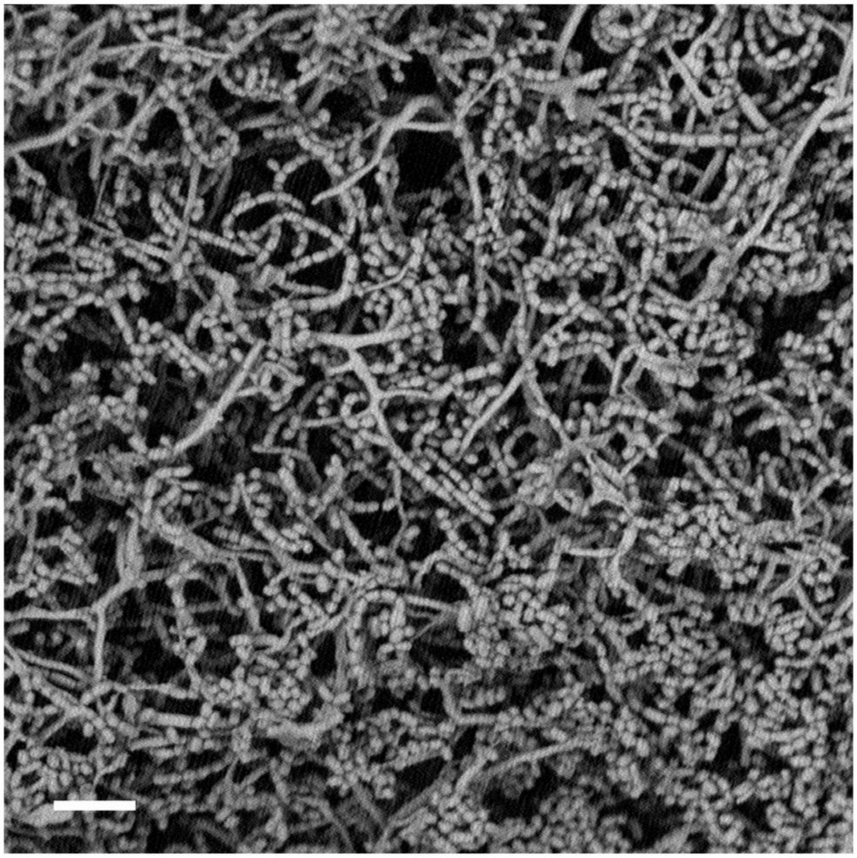

Figure S1. Scanning electron micrograph of the *ylmDE* double mutant of *S. coelicolor* M145.

Normal sporulation is restored to *ylmE* mutant by the additional deletion of *ylmD*. Cultures were grown on SFM agar plates for 5 days at 30^0C. Bar, 5 µm.

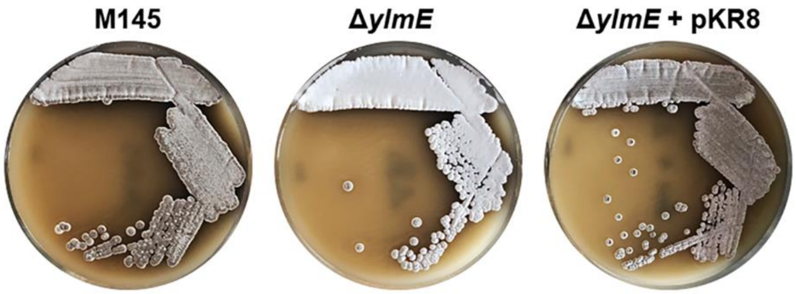

Figure S2. Complementation of *ylmE* mutants via *dcw* cluster. Wild type M145, *ylmE* null mutant and *ylmE* mutant containing construct pKR8 were grown on SFM agar plates for 5 days at 30^{0}C. Introduction of pKR8 which harboring *murD*, *ftsW*, *murG*, *ftsQ*, *ftsZ*, *ylmD*, *ylmE*, *sepF*, *sepG* and *divIVA*, fully restored sporulation of *ylmE* null mutant.

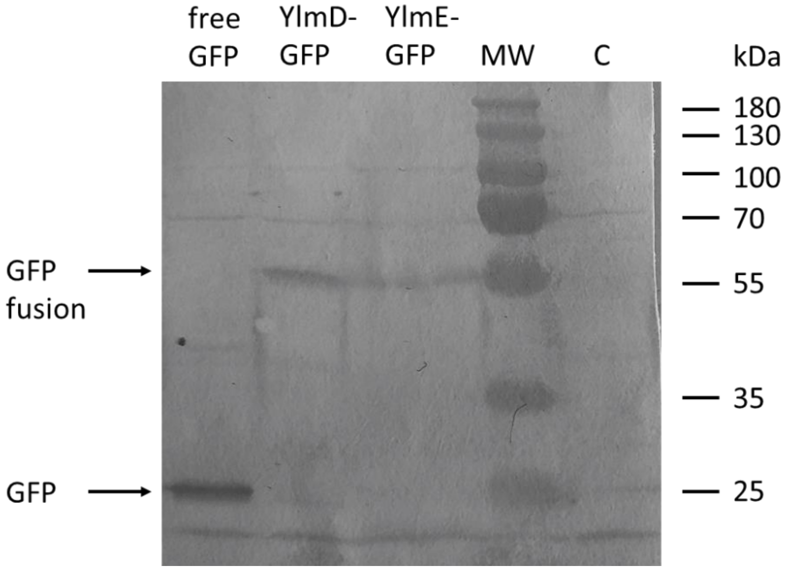

Figure S3. Western blot analysis with anti-GFP antibodies. Total protein was extracted from biomass collected from cellophane overlaid SFM agar plates after 6 days of growth at 30°C. Freely mobile eGFP was detected in protein extract from M145 expressing eGFP under the

control of the constitutive *gap* promoter. GFP antibodies identified full length YlmD-eGFP and YlmE-eGFP fusion proteins, which were expressed in strain GAL 50 and GAL49, respectively. No GFP was identified in *S. coelicolor* M145 with control plasmid (C). MW, prestained protein protein marker (approximate molecular weight in kDa).

References

1 Sambrook, J., Fritsch, E. & Maniatis, T. *Molecular cloning: a laboratory manual.*, (New York: Cold spring harbor laboratory press, 1989).

2 MacNeil, D. J. *et al.* Analysis of *Streptomyces avermitilis* genes required for avermectin biosynthesis utilizing a novel integration vector. *Gene* **111**, 61-68, https://doi.org/10.1016/0378-1119(92)90603-M (1992).

3 Kieser, T., Bibb, M. J., Buttner, M. J., Chater, K. F. & Hopwood, D. A. *Practical Streptomyces genetics.*, (John Innes Foundation, 2000).

4 Grantcharova, N., Lustig, U. & Flärdh, K. Dynamics of FtsZ assembly during sporulation in *Streptomyces coelicolor* A3(2). *J. Bacteriol.* **187**, 3227-3237, https://doi.org/10.1128/JB.187.9.3227-3237.2005 (2005).

5 Vara, J., Lewandowska-Skarbek, M., Wang, Y. G., Donadio, S. & Hutchinson, C. R. Cloning of genes governing the deoxysugar portion of the erythromycin biosynthesis pathway in *Saccharopolyspora erythraea* (*Streptomyces erythreus*). *Journal of bacteriology* **171**, 5872-5881, (1989).

6 Larson, J. L. & Hershberger, C. L. The minimal replicon of a streptomycete plasmid produces an ultrahigh level of plasmid DNA. *Plasmid* **15**, 199-209, https://doi.org/10.1016/0147-619X(86)90038-7 (1986).

7 Fedoryshyn, M., Welle, E., Bechthold, A. & Luzhetskyy, A. Functional expression of the Cre recombinase in actinomycetes. *Applied microbiology and biotechnology* **78**, 1065-1070, https://doi.org/10.1007/s00253-008-1382-9 (2008).

8 Ramijan, K. *et al.* Reversible metamorphosis in a bacterium. *bioRxiv*, 094037, (2016).
